# Strengthening the Health System as a Strategy to Achieving a Universal Health Coverage in Underprivileged Communities in Africa: A Scoping Review

**DOI:** 10.3390/ijerph19010587

**Published:** 2022-01-05

**Authors:** Anelisa Jaca, Thobile Malinga, Chinwe Juliana Iwu-Jaja, Chukwudi Arnest Nnaji, Joseph Chukwudi Okeibunor, Dorcas Kamuya, Charles Shey Wiysonge

**Affiliations:** 1 Cochrane South Africa, South African Medical Research Council, Cape Town 8000, South Africa; thobilemalinga@outlook.com (T.M.); chuksmannaji@yahoo.com (C.A.N.); charles.wiysonge@mrc.ac.za (C.S.W.); 2 Department of Nursing and Midwifery, Faculty of Medicine and Health Sciences, Stellenbosch University, Cape Town 8000, South Africa; chinwelolo@gmail.com; 3 School of Public Health and Family Medicine, University of Cape Town, Cape Town 8000, South Africa; 4 World Health Organization Regional Office for Africa, Brazzaville, Congo; okeibunorj@who.int; 5 Department of Health Systems and Research Ethics, KEMRI-Wellcome Trust Research Programme, Nairobi 43640-00100, Kenya; DKamuya@kemri-wellcome.org; 6 Division of Epidemiology and Biostatistics, Faculty of Medicine and Health Sciences, Stellenbosch University, Cape Town 8000, South Africa

**Keywords:** universal health coverage, health systems, low- and middle-income countries, African countries

## Abstract

Universal health coverage (UHC) is defined as people having access to quality healthcare services (e.g., treatment, rehabilitation, and palliative care) they need, irrespective of their financial status. Access to quality healthcare services continues to be a challenge for many people in low- and middle-income countries (LMICs). The aim of this study was to conduct a scoping review to map out the health system strengthening strategies that can be used to attain universal health coverage in Africa. We conducted a scoping review and qualitatively synthesized existing evidence from studies carried out in Africa. We included studies that reported interventions to strengthen the health system, e.g., financial support, increasing work force, improving leadership capacity in health facilities, and developing and upgrading infrastructure of primary healthcare facilities. Outcome measures included health facility infrastructures, access to medicines, and sources of financial support. A total of 34 studies conducted met our inclusion criteria. Health financing and developing health infrastructure were the most reported interventions toward achieving UHC. Our results suggest that strengthening the health system, namely, through health financing, developing, and improving the health infrastructure, can play an important role in reaching UHC in the African context.

## 1. Introduction

Universal health coverage (UHC) is defined as people having access to quality healthcare services (e.g., treatment, rehabilitation, and palliative care) they need, irrespective of their financial status [1]. Unfortunately, access to quality healthcare services continues to be a problem for many individuals and communities in LMICs. Most people residing in LMICs do not have access to quality healthcare services and financial risk protection [1,2]. In the context of LMICs, quality implies access to timely, safe, affordable, and effective treatments without discrimination by socioeconomic status [3,4]. In Africa, access and coverage of essential healthcare services is poor due to various challenges, namely, the shortage and inequitable distribution of healthcare providers; service capacity (e.g., stock-outs of medicines and healthcare equipment, inadequate number of health professionals); late payment to health professionals; physical accessibility to primary health centers; poor access to health information or poorly developed health information systems; and population growth [5,6]. All these lead to demotivation of health professionals and thus poor health service to patients [5]. UHC can be achieved through focusing on three main factors, firstly, providing financial support to people attending healthcare services. Spending a lot of money to access healthcare can result in people cutting down on their basics needs such as food, clothing, and children’s education. Additionally, people may decide not to use services, due to costs related (e.g., consultations, medicines, and laboratory tests) to healthcare. Providing financial support to people who cannot afford healthcare implies that countries must reduce financial costs incurred while seeking healthcare. Financial costs can be reduced through strategies such as the national health financing, social health insurance, and tax-based financing of healthcare.

Secondly, UHC can be achieved through widening population coverage: for example, health insurance companies that cover their employees could also cover their employees’ relatives and dependents while the government covers the underprivileged. Poor people residing in middle- and high-income countries could also be covered through tax payers. Health ministries could also try to procure funds enough to cover a large population as far as healthcare services are concerned. Thirdly, UHC can be achieved through improving the scope and quality of services (service coverage) [1,2].

Health system strengthening, which involves improving a system’s performance to restore and maintain people’s health, is another significant way to achieving UHC [7,8]. Health system strengthening contributes to sustainable development goals (SDGs) in several ways, i.e., reducing poverty, providing equitable health outcomes, and improving well-being [9]. UHC depends on the availability, accessibility, and capacity of healthcare facilities and healthcare workers to deliver quality healthcare services [9]. However, access to quality healthcare services continues to be a problem for most people in low- and middle-income countries (LMICs). Most people do not have access to basic healthcare services globally; many of those reside in LMICs [1]. In Africa, accessibility and coverage of adequate supply of essential healthcare services is low due to financial constraints caused by factors such as out-of-pocket expenditures, especially among women and underprivileged individuals [10]. Other factors contributing to the challenge of accessibility involves distance to health facilities in rural areas and unavailability of quality of services, equipment, essential drugs, and healthcare workforce. People residing in rural areas travel long distances to seek healthcare services, which can be very costly [11,12]. The fragile health infrastructure in the continent has raised doubts as to whether this region can provide equitable access to healthcare to its communities. There are also doubts regarding whether African countries can deliver equitable access to healthcare to disadvantaged populations. Therefore, the WHO recommends developing health systems to ensure that people are covered. This study focuses on the supply of healthcare services (e.g., through strengthening the healthcare system) since the health infrastructure, which is a basic need for achieving UHC, is poor in the African continent. The poor health system in turn negatively affects service delivery, and therefore improving this area would ensure the delivery of quality healthcare in the continent.

It is therefore imperative for the African continent to focus on building and improving the health infrastructure, e.g., by reducing out-of-pocket payment and increasing population and service coverage. This can be done by sourcing funding and enhancing healthcare workforce as the most cost-effective way to ensure access to primary healthcare for all [13]. The aim of this paper was to conduct a scoping review to map out the strategies used to strengthen the health system to achieve UHC in Africa among underprivileged communities. This review focuses on disadvantaged populations as they are among the most excluded individuals and groups in terms of access to healthcare services. There are lessons that can be derived from countries that have been successful in implementing strategies to inform UHC, thereby improving equitable access to care. To the best of our knowledge, no review has been conducted to report on the strategies aimed at improving the health system in the African setting, specifically where the marginalized population is concerned.

## 2. Materials and Methods

This scoping review was conducted to map out the crucial interventions and bring together the existing evidence on the interventions used to strengthen the health systems as a strategy for achieving UHC in Africa. We employed a predefined protocol as per the Arksey and O’Malley methodological outline to carry out this scoping review [14]. This outline included identifying the research question, searching for relevant studies, selecting studies, charting and collating data, summarizing, and reporting results. This scoping review was reported as per the Preferred Reporting Items for Systematic reviews and Meta-Analyses extension for Scoping Reviews (PRISMA-ScR) Checklist [14].

### 2.1. Defining the Research Question

The WHO put up in place a framework for action on strengthening health systems, based on six health system building blocks: (i) service delivery, (ii) health workforce, (iii) health information systems, (iv) access to essential medicines, (v) financing, and (vi) leadership/governance [15]. Health system strengthening was defined as improving these six health systems building blocks to obtain equitable and quality healthcare services. This scoping review was therefore performed to report the strategies used to strengthen health systems in the African setting. The research question “What are the strategies used to strengthen the health system in order to achieve UHC in Africa?” was framed using the Population, Concept, and Context (PCC) element (Table 1).

### 2.2. Inclusion Criteria

We included case control, cross-sectional, qualitative, quantitative, review, and scenario-based studies that investigated the areas where health systems are strengthened as a means of achieving UHC in the African context. These areas included integrating health systems of different countries, financing health systems, enhancing staff members (e.g., investing in nurses), fairly distributing healthcare facilities, improving access to medicine, introducing insurance subsidies, and enhancing a political will among political leaders. Eligible population included evidence producers (health researchers) and evidence users (i.e., health policy makers, beneficiaries of UHC, non-government organizations, and healthcare providers). The outcome measures included UHC outcomes involving healthcare service coverage, access to healthcare services for all populations, and financial risk protection. Country reports compiled by the World Bank, WHO, and USAID on the progress and challenges to achieving UHC in Africa were also included in this review. The data collection tool for the World Bank’s Universal Health Coverage Studies Series (UNICO studies) was made up of “Nuts & Bolts” questionnaire with nine components, which had 329 questions. The components collected information on a country’s health system, selected programs, public financing of the program, cost containment, and the information environment. Data collection was conducted and technical reports were written for each country [16].

### 2.3. Search Strategy

One author (A.J.) developed a search strategy with input from CAN and CIJ and performed a comprehensive literature search in PubMed on 10 July 2020 and Scopus on 14 July 2020 with no language or date restrictions. We did a literature search on two databases since they are deemed sufficient when conducting a scoping review. In our search, we combined the key terms such as “Comprehensive Health Insurance” (MeSH Terms) OR “Universal Coverage” (MeSH Terms) OR “Insurance Coverage” (MeSH Terms) OR “Insurance, Hospitalization” (MeSH Terms) OR “Single-Payer System” (MeSH Terms) OR “Prepaid Health Plans” (MeSH Terms) OR “Not-For Profit Insurance Plans” (MeSH Terms) OR “Insurance, Health” (MeSH Terms) OR “Universal Health Coverage” (MeSH Terms) (Table 2). We included non-Sub-Saharan African countries in our search as this study is investigating strategies to increase UHC in Africa as a whole. After having run and tested the search in PubMed, A.J. reviewed the first 20 search results to determine if the studies produced were related to the topic being investigated. When agreement was reached about the search strategy, the first 100 records were screened by A.J. on concepts potentially eligible for inclusion in the search string. We selected search terms related to the population (e.g., health policy makers, healthcare providers, health insurance coverage), concept (universal health coverage), and context (African countries) (Table 1). A.J. then developed a search query using Boolean operators AND and OR for PubMed and then applied it to Scopus. The authors did not employ the services of a librarian in developing the search strategy as CAN and CIJ have vast experience in searching for studies.

### 2.4. Selection of Studies Relevant to the Research Question

Selection of studies was independently conducted by two authors (A.J. and T.M.) based on the definition of the research question derived from the PCC concept. Final decisions about eligible studies were achieved through meetings by A.J. and T.M., where the two authors did not encounter any challenges and reservations related to the selection process.

### 2.5. Extraction and Charting the Results

Two authors (A.J. and T.M.) independently extracted data using a specially designed data extraction form. Information was extracted on author name, the year in which the study was published, the country where the study was conducted, study design, participants, and the key messages derived from the results. The two authors compared extracted data and resolved differences through discussion and consensus. Key messages, based on the results of each study, were summarized and reported using narrative descriptions through consensus between the two authors (A.J. and T.M.). Given the nature of our research question and that most of the studies included were qualitative or descriptive in nature, we summarized the main findings using a narrative synthesis. We grouped the interventions that emerged from included studies into four main themes, i.e., health workforce: developing and improving health infrastructure; health information systems: health technology; access to essential medicines; and health financing.

### 2.6. Patient and Public Involvement

This study used publicly available data and thus did not involve any patients and the public.

## 3. Results

**Figure 1:**
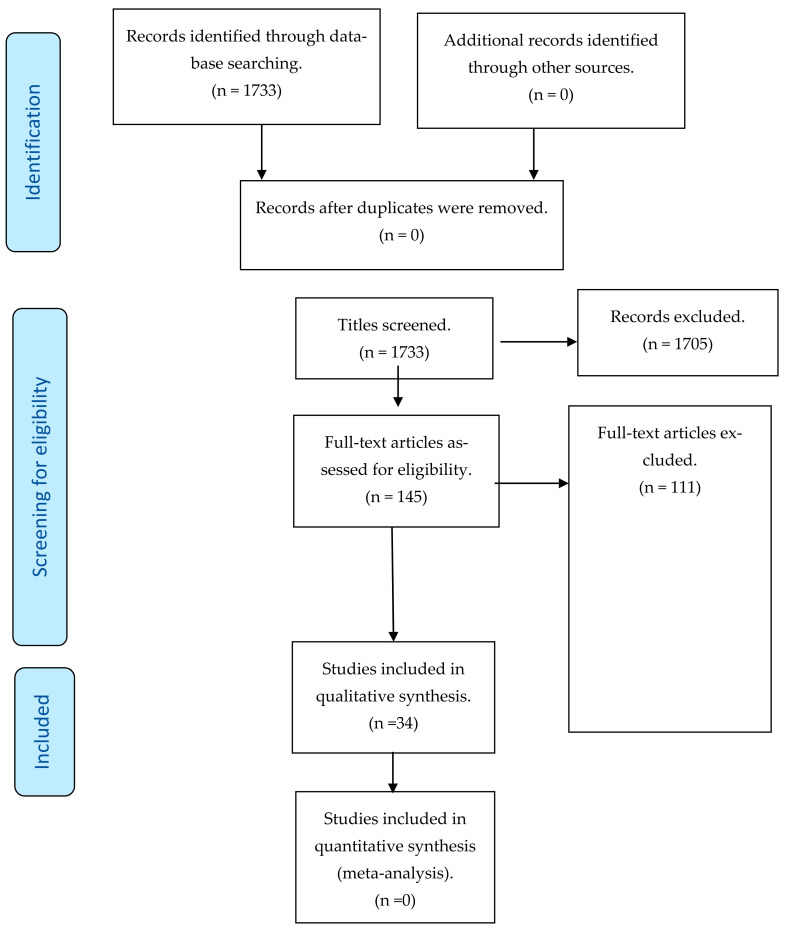
Study selection process for the scoping review.

The literature search yielded a total of 1733 records, of which 894 were retrieved from PubMed and 839 from Scopus. The search outputs from PubMed and Scopus were imported into Mendeley software to identify duplicates. There were, thus, no duplicates seen across the two databases. We therefore screened a total number of 1733 of abstracts and titles, of which 152 were potentially eligible (Figure 1). Of these potentially eligible papers, we could access the full texts of 145 journal articles written in English. These articles were then assessed for eligibility and 34 met the inclusion criteria (Table 3). Reasons for exclusion included wrong study designs, not investigating the strategies to increase UHC, or studies not conducted in the African setting.

### 3.1. Characteristics of Included Studies

#### 3.1.1. Setting and Publication Dates

Of the 34 included studies, the majority were conducted in Nigeria (6 studies), Ethiopia (5 studies), South Africa (5 studies), and Tanzania (5 studies). Settings including Ghana (4 studies), Malawi (3 studies), Rwanda (3 studies), Kenya (2 studies), Senegal (2 studies), and Zambia (2 studies) produced the least publications on UHC. Moreover, the countries Algeria, Botswana, Burkina Faso, Egypt, Democratic Republic of Congo, Madagascar, Seychelles, and Zimbabwe had only one study each (Table 4). The 34 included studies were conducted between 2001 and 2020 and investigated how health system strengthening strategies can achieve UHC. These strategies include health financing; contracting out delivery of primary health care services; retention of qualified healthcare workers in rural areas; strengthening the evidence ecosystem; introducing African traditional medicine (ATM) and traditional health practitioners (THPs); providing access to medicines; improving quality of primary healthcare; health technology assessment (HTA); and expanding the primary care network and health infrastructure (Table 3).

#### 3.1.2. Study Designs

Of the included studies, there were 24 cross-sectional, 4 case studies, 2 qualitative studies, 1 qualitative and quantitative study, and a scenario analysis

#### 3.1.3. Participants

The type of participants included in the eligible studies were classified into three themes, i.e., health providers, policy makers, and patients.

##### Health Providers

These included healthcare workers [18]; nurse facility heads and district nurse supervisors [49]; doctors, nurses, midwives, and superior technicians in anesthesiology [24]; healthcare providers from the public and private sector [27,43]; and traditional and conventional health practitioners [23].

##### Policy Makers

Policy makers involved stakeholders from the health and financial sectors [17]; providers of services under the formal sector social health insurance [18]; district councils across the country [19]; governments, implementers, drone providers, and funders of healthcare [41]; politicians and donor officials [21]; enrollees’ medical care utilization [47]; actors and policy processes [22]; health system governance and community health worker programs [42]; national UHC legislation that promote universal access to medicines [26]; decision makers, academic researchers, civil society organizations, community-based organizations, development partners, health professional organizations [29]; private health systems; Ministry of Finance [30]; and a range of political contexts [32].

##### Patients/Consumers

Household members [25,28,31,33,48]; people with access to a private subsidized health insurance program [34]; community members [46]; HIV+ pregnant women [36]; pregnant women [37]; poor women who had given birth [38]; community residents [39]; males and females aged 23 to 59 years [45]; patients exiting healthcare facilities [40].

### 3.2. Interventions to Increase Universal Health Coverage

This scoping review reports the different strategies (derived from the included studies) to strengthen the health system for attaining UHC in Africa. These strategies include those that fall under capacity building in health facilities, as well as developing and improving health infrastructure (e.g., training, supervising, and retaining healthcare workers, developing healthcare facilities) [19,24,27,30,35,42,49]; health information systems (health technology assessment) [29,41]; access to essential medicines (access to medicines and introducing African traditional medicine and traditional health practitioners into health systems) [23,26]; and health financing [17,18,20,21,22,25,28,31,32,33,34,36,37,38,39,40,43,44,45,46,47,48,50].

#### 3.2.1. Health Workforce: Developing and Improving Health Infrastructure

It was reported that UHC can be attained by developing and improving the health infrastructure in Ethiopia, Senegal, Seychelles, South Africa, Tanzania, and Zambia [19,24,27,30,35,42,49]. These strategies include increasing leadership capacity and management skills of facility managers, increasing healthcare worker force, and upgrading primary healthcare facilities. Strengthening health systems by supporting community health worker programs and keeping qualified healthcare workers in rural areas were also reported. Moreover, improving the quality of primary healthcare through training and supervision and expanding the primary healthcare network were also mentioned.

#### 3.2.2. Health Information Systems: Health Technology 

Two of the included studies (case and cross-sectional studies) reported the use of technology such as bi-directional transport drones to strengthen health systems. The adoption of health technology assessment as an instrument to support UHC-related decision making was also reported [29,41]. The two studies indicated that more evidence needs to be generated to validate the impact of these strategies on strengthening health systems [20,32].

#### 3.2.3. Access to Essential Medicines

Two of the included studies reported that access to medicine, and African traditional medicine (ATM) can also be used as strategies to strengthen the health system [23,26].

#### 3.2.4. Health Financing

Of the 34 studies included, 23 focused on assessing the role of health financing, as a health system strengthening strategy in Botswana, Egypt, Ethiopia, Democratic Republic of Congo, Gabon, Ghana, Kenya, Malawi Nigeria Rwanda, South Africa, Tanzania, Zambia, and Zimbabwe [17,18,20,21,22,25,28,31,32,33,34,36,37,38,39,40,43,44,45,46,47,48,50]. These studies investigated and reported various ways in which finances could be enhanced, i.e., through household out-of-pocket payments, health insurance, and attracting donors and taxes. People either postponed or did not seek care when needed due to financial constraints hence financing the health system is important to achieve access to healthcare [20]. One study stated that older individuals, those who are far from health facilities, with chronic diseases and low socioeconomic status are not likely to attend healthcare services due to a high out-of-pocket expenditure [25].

Other included studies reported that social and community health insurance schemes could play a huge role in achieving UHC [44]. One study stated that health insurance is merely one of many future policy options that will help achieve UHC [21]. Other studies reported that health insurance plays a vital role and improves medical care use and reduces out-of-pocket expenditures [18,31,44,47]. Another study reported that community-based health insurance member households are more likely to utilize outpatient care than their non-member counterparts [33]. Contracting out the delivery of primary healthcare services to the private sector was also another avenue of alleviating the financial burden of the health system [22,32,48,50].

## 4. Discussion

Universal health coverage is a strategy that was developed to address the challenges encountered by different states in providing access to quality healthcare services without costs [1]. The nature of our review question warranted the use of a narrative synthesis method, as statistical meta-analysis was not feasible. A narrative synthesis is a suitable approach for the analysis of findings from multiple studies, especially where most of the results are reported qualitatively, as in the studies included in the present review. This type of approach uses themes and words to summarize and explain the findings from the included studies. Therefore, this review derived themes from the included studies; these themes were guided by and structured according to the six building blocks of the WHO Health System Strengthening Framework, namely, (i) service delivery, (ii) health workforce, (iii) health information systems, (iv) access to essential medicines, (v) financing, and (vi) leadership/governance. The current study therefore reports on four key interventions, namely, developing and improving health infrastructure, health technology systems, access to essential medicines, and health financing; these interventions fall under some of the six building blocks of the WHO Health System Strengthening Framework. The included studies mentioned these as the strategies that countries may focus on to achieve UHC.

Previous reviews were conducted in Africa [37] and low- and middle-income countries (LMICs) outside Africa around UHC [2,52,53]. These previously conducted investigations focused on health financing and suggested that financial constraints are a significant limitation to accessing quality healthcare services in LMICs. This suggests that people who cover their own costs for health services are mainly incapable of getting the services they require. Therefore, moving toward UHC requires strengthening health systems and vigorous financing structures in all countries [54].

Furthermore, to attain UHC, investing in healthcare infrastructure, e.g., increasing and retaining healthcare workers in Africa is essential. The WHO stated that more than 18 million additional health workers are required by 2030 to achieve UHC and so there is a need to supply healthcare workers in low- and lower-middle-income countries. The extent of improving health system infrastructure, such as training and retaining healthcare workers, developing healthcare facilities, and ensuring access to basic healthcare, is critical to attaining UHC, especially in low-resource regions such as Africa. One previous review also inferred that both public and private stakeholders need to invest in the training of healthcare workers and in creating positions in the health fraternity [52]. There were no similar reviews that focused on how health technology and access to essential medicines could drive UHC in the continent. Thus, reviews need to be conducted to map out how health technology and access to essential medicines could drive UHC in Africa.

### 4.1. Country Reports on Achieving UHC in Africa

In this study, we saw it fit to also retrieve and report on country reports around UHC. The reports included in this review stated ways in which the UNICO countries, with Ethiopia, Ghana, Kenya, Nigeria, South Africa, and Tunisia being the only mentioned African countries, could achieve UHC. In Africa, areas that should be focused on to improve UHC include effective financial protection, people-centered services, targeting the poor and the marginalized, and strengthening health security. Population (which individuals are covered?), services (which services are covered?), and cost sharing (what costs are covered?) should be the focus or the start toward achieving UHC. The World Bank, through UNICO, compiled reports from developing countries that have committed to achieving UHC following a bottom-up approach, which focuses on underprivileged and susceptible people. These reports described the experiences that different countries have had. This was based on a methodical data collection effort that aimed to give a detailed account on how they are implementing UHC. This was done to gain insight into policy makers and other stakeholders whose aim is to achieve UHC. The countries were selected for the valuable efforts they have made in the past, to expand coverage of healthcare services, focusing on the poor. These countries have not yet reached UHC; however, they provide important lessons from which to learn about UHC. These countries reported the following two broad approaches to achieving UHC [16,55].

The first approach is the “supply-side programs,” channels investments to increase the size of services that are provided. This can be done through acquiring finances meant to recruit staff and develop public clinics. The second broad approach entails “demand-side programs.” These programs are aimed at identifying and enrolling their target populations and cover healthcare services through output-based payments. Since the underprivileged do not have enough money to cover the healthcare services they seek, two African countries, i.e., Ethiopia and South Africa considered a national health insurance (NHI) program in 2013. These countries envisaged that the NHI program would coexist with the services of the Ministry of Health [55].

### 4.2. Progress and Challenges of UHC in Africa

#### Government Spending on Health Has Increased

Two African countries, i.e., Lesotho and Nigeria reported a GDP increase, with Nigeria reaching 10.5% and Lesotho 60.5%. Several countries use their own funds to finance healthcare services due to limited funding or income. This lack of funding or income resulted in many different African states increasing their budgets by 15% to fund healthcare services as per the 2001 Abuja target. However, only four African countries (i.e., Ethiopia, Gambia, Malawi, and Swaziland) reached and maintained the Abuja target by 2014 [56].

### 4.3. Growth in Development Assistance for Health

There has been a swift growth concerning the development assistance for health (DAH) in Africa. This development is a result of spending too much money to manage HIV/AIDS and malaria. From 1990 to 2010, the money dedicated toward managing HIV/AIDS was reported to have increased from 7% to 54% and 1% to 13% for malaria. Therefore, the total funds spent on healthcare services in Africa ranged from 20% to 35% between the years 2000 and 2014 [56].

### 4.4. Service Delivery Capacity Has Expanded

A substantial number of healthcare workers is vital for a country to maintain access to quality healthcare services. In Africa, there has been a lack of skilled healthcare workers, which is a serious challenge to obtaining UHC. The lack of healthcare workers such as doctors, nurses, and midwives in Africa is due to insufficient experience in this sector [56].

### 4.5. Non-State vs. State Healthcare Workers in Achieving UHC

There is proof that contracting services outside a country works very well in providing healthcare services. Although it is challenging for states to maintain these types of corporations, republics continue to contract out some services related to healthcare, e.g., transport and medical waste disposal to other foreign countries [56].

### 4.6. Essential Medicines and Technologies

Although little progress has been made regarding achieving UHC in some African counties, access to medicines continues to be problematic. The challenges to accessing medicines in the African continent are related to limited funding, improper management at pharmaceutical companies, and lack of information. Another problem is the fact that health insurance companies do not cover some medicines and therefore disadvantaged individuals are always left out in the cold [56].

### 4.7. Strengths and Limitations of the Study

We note that this study may have some limitations and one that stands out involves searching two databases for eligible studies. Therefore, we may have missed more primary studies that we could have retrieved from other electronic databases, including gray literature. In addition, we could not access full-text articles of seven of our studies; therefore, we acknowledge that this may have limited the number of studies included in this study. In future research, it would be of benefit to conduct a comprehensive literature search on other databases to possibly retrieve more literature that will contribute to the study being updated. Another significant limitation is that this review focused on underprivileged settings, which implies that the results cannot be generalized throughout the continent. However, the results reported in this review are innovative, and according to our knowledge, the review is the first to synthesize evidence on strategies to strengthen the health system to achieve UHC among underprivileged communities in Africa. Due to the nature of the study designs included in this review, we could only conduct a narrative synthesis and not a meta-analysis. This is also a limitation in this study.

## 5. Conclusions

UHC, which entails ensuring that all individuals have access to quality healthcare without any financial difficulties, forms part of the 2030 agenda for sustainable development goals. Hence, implementing different strategies such as strengthening the health system (e.g., by building health infrastructure and retaining healthcare worker force), and providing financial assistance to achieve UHC in underprivileged communities in the African continent, is imperative. The current study maps out and reports the strategies from the included studies to strengthen the health system (i.e., developing, and improving the health infrastructure, health technology, access to essential medicines and health financing). The studies included in this review reported that the above-mentioned strategies could play an important role toward reaching UHC in low- and middle-income settings in Africa. The studies included in this review have stated ways in which health financing can be achieved, e.g., introducing health insurance schemes, reducing out-of-pocket expenditure and taxes, and integrating the private and public sectors. In addition, a few of the studies included also reported that the cost-effective way of strengthening the health system is improving the health infrastructure by retaining and employing more healthcare workers in the African continent. The interventions reported by the studies included in this review can be generalized to other low- and-middle income countries in Africa, since they also represent underprivileged communities. A lot of research has been conducted on how health financing could attain UHC, but there is still a lot of work to be done to report on how developing health infrastructure and increasing access to essential medicines can achieve UHC in the continent.

## Figures and Tables

**Table 1:** PCC element for defining the eligibility criteria of the studies for the research question.

Population	Concept	Context
Health policy makers, healthcare providers, health facilities, government and non-government organizations (e.g., health insurance companies)	Access to healthcare services or UHC	Africa

**Table 2:** PubMed and Scopus search strategies.

Search #	Search String
#1	“Comprehensive Health Insurance” OR “Universal Coverage” OR “Insurance Coverage” OR “Insurance, Hospitalization” OR “Single-Payer System” OR “Prepaid Health Plans” OR “Not-For Profit Insurance Plans” OR “Insurance, Health”) OR “Universal Health Coverage”
#2	implement* OR facilitat* OR enabl* OR resist* OR promot* OR applica* OR integrat* OR utiliz* OR utilis* OR success* OR access* OR accept* OR lesson* OR experienc* OR scale-up OR roll-out OR sustainab*
#3	“Health systems” OR “Health facility” OR “Health care services” OR “Health services” OR “Healthcare services” OR “Healthcare workers” OR “Health care workers” OR “Healthcare providers” OR “Health care providers”
#4	Algeria OR Angola OR Benin OR Botswana OR Burkina Faso OR Burundi OR Cameroon OR “Cape Verde” OR “Central African Republic” OR Chad OR “Democratic Republic” of Congo OR “Republic of Congo” OR “Cote d’Ivoire” OR Djibouti OR Egypt OR “Equatorial Guinea” OR Eritrea OR Ethiopia OR Gabon OR Gambia OR Ghana OR Guinea OR Guinea Bissau OR Kenya OR Lesotho OR Liberia OR Libya OR Madagascar OR Malawi OR Mali OR Mauritania OR Mauritius OR Morocco OR Mozambique OR Namibia OR Niger OR Nigeria OR Reunion OR Rwanda OR “Sao Tome and Principe” OR Senegal OR Seychelles OR “Sierra Leone” OR Somalia OR “South Africa” OR “South Sudan” OR Sudan OR Swaziland OR Tanzania OR Togo OR Tunisia OR Uganda OR Zambia OR Zimbabwe
#5	#1 AND #2 AND #3 AND #4

**Table 3:** Characteristics of included studies.

Author, Year	Population	Intervention to UHC	Aim of the Study	Outcome	Study Design	Setting
Abdulmalik, 2019 [17]	Health and financial sectors stakeholders.	Financing support and increasing HCW force.	To review the health and socioeconomic contexts of Nigeria as well as to generate strategies for sustainable mental health financing that will be feasible, within the specific context of the country.	Financing strategies: governance and leadership, financing generation, and financing pooling.	Cross-sectional study	Nigeria
Ebunoha, 2019 [18]	Consumers and providers of the Formal Sector Social Health Insurance Programme and healthcare workers.	Financing support and increasing HCW force.	To understand peoples’ perceptions of the Formal Sector Social Health Insurance Programme. To understand the prevalence of out-of-pocket among enrollees of the FSSHIP, which will provide an evidence base on the financial risk protection that the scheme currently provides.	Nigeria needs to show a strong commitment to achieving UHC by involving the informal sector in the social health insurance scheme through awareness campaign about the benefits of NHIS.	Cross-sectional study	Nigeria
Kapologwe, 2020 [19]	Primary health facilities.	Developing and upgrading infrastructure of primary healthcare facilities.	To understand the public sector’s efforts to improve the infrastructure of primary health facilities between 2005 and 2019.	In this study only 17.4% of 115 health centers had facilities for offering blood transfusion services and 33% (1673) of health facilities had piped water and 5.1% had landline telecommunication system.	Cross-sectional study	Tanzania
Laokri, 2018 [20]	Household and health facility representatives.	Health financing through household out-of-pocket payments.	To gain a deeper understanding of the poverty impact of using essential care services in selected provinces in the DRC. We sought to inform the evidence base regarding the policy and technical challenges inherent to developing financial risk protection strategies.	Those who did not seek care when needed reported financial constraints as the major reason for postponing or foregoing care. Wealth-related inequities were found in service and population coverage and in out-of-pocket payment for outpatient care.	Cross-sectional study	Democratic Republic of Congo
Lavers, 2019 [21]	Politicians, policy makers, and donor officials.	Health financing through social health insurance.	To examine the political drivers of the adoption and evolution of state health insurance based on 28 key informant interviews conducted between 2015 and 2018 with politicians, policy makers, and donor officials.	The government noted insurance as a possible source of finance in the 1993 Health Policy and 1998 Healthcare Financing Strategy (HCFS). Nonetheless, health insurance was merely one of many future policy options, without a clear implementation strategy.	Cross-sectional study	Ethiopia
Maluka, 2018 [22]	Actors, policy processes, and content.	Contract out the delivery of primary health care services.	To report on the design and implementation of service agreements (SAs) between local governments and NSPs for the provision of primary healthcare services in Tanzania. It examines the actors, policy process, context, and policy content that influenced how the SAs were designed and implemented.	Delays in reimbursements, limited financial and technical capacity of local government authorities, and lack of trust between the government and private partners affected the implementation of the contractual arrangements.	Cross-sectional study	Tanzania
Kasilo, 2019 [23]	Traditional and conventional health practitioners.	African traditional medicine (ATM) and traditional health practitioners (THPs).	To report the regional status of African traditional medicine and traditional health practitioners (THPs) using information from the survey conducted in 47 countries in sub-Saharan Africa. The survey focused on the nature and level of collaboration between THPs and community-based health plans concerning research, training, treatment, and care.	The significant progress made by some African countries in the development and integration of ATM and THPs into the national health systems provides lessons that can be harnessed to improve access to quality health services and subsequently contribute to the attainment of UHC. The research infrastructure and mechanisms in place provide a platform for further development of efficacious and affordable ATM.	Cross-sectional study	47 countries in the WHO African region
Nagai, 2017 [24]	Doctors, nurses, midwives, and superior technicians in anesthesiology.	Retention of qualified healthcare workers in rural areas.	To identify the determining factors for the retention of qualified human resources for health in rural areas, and to explore an effective and feasible policy that the Ministry of Health could implement in the member countries.	Improved human resources for health (HRH) management, e.g., the transparency of human resource management by the MoH, was identified as a pre-condition of any policy implementation related to HRH. This factor can be considered in other countries struggling to retain healthcare workers in rural areas.	Cross-sectional study	Senegal
Nakovics, 2020 [25]	Households.	Financial support (out-of-pocket expenditure).	To investigate out-of-pocket expenditure (OOPE) on curative healthcare services and their determinants in rural Malawi, a country that has consistently aimed at providing free healthcare services.	Our findings indicate that a formal policy commitment to providing free healthcare services is not enough to guarantee widespread financial protection and that additional measures are needed to protect particularly vulnerable population groups.	Cross-sectional study	Malawi
Perehudof, 2019 [26]	National UHC legislation that promote universal access to medicines.	Access to medicines.	To identify and compares legal texts from national UHC legislation that promote universal access to medicines in the legislation of 16 mostly LMICs.	National policies, particularly for pharmaceuticals, health, and intellectual property, can instruct the development of health law or substitute it entirely by directing state policies and programming. Future research should investigate the content, implementation, and impact of national policies in relation to access to medicines as part of the right to health.	Cross-sectional study	Algeria, Ghana, Nigeria, Rwanda, South Africa, Tanzania
Renggli, 2019 [27]	Healthcare providers from the public and private sector.	Improving quality of primary healthcare through enhanced routine supportive supervision.	To improve quality of primary healthcare through enhanced routine supportive supervision.	The results showed that the new approach managed to address quality issues that could be solved either solely by the healthcare provider, or in collaboration with the council. The new approach was able to improve and maintain crucial primary healthcare quality standards across different health facility level and owner categories in various contexts.	Cross-sectional study	Tanzania
Shamu, 2016 [28]	Households.	Health financing.	To assess the utilization of healthcare services and benefits at different levels of care by different socioeconomic groups.	Our results showed that richer people disproportionately benefited from public health subsidies overall, particularly at secondary and tertiary levels, which receive more funding and provide a higher level of care.	Cross-sectional study	Zimbabwe
Uzochukwu, 2020 [29]	Decision makers, policy makers, academic researchers, civil society organizations, community-based organizations, development partners, and health professional organizations.	Health technology assessment (HTA) is an effective tool to support priority setting and generate evidence for decision making especially en route to achieving UHC.	To assess the capacity needs, policy areas of demand, and perspectives of key stakeholders for evidence-informed decision making in Nigeria, where health technology assessment is still new.	Public health programs, medicines, and vaccines were the three main technology types that would especially benefit from the application of HTA. The perceived availability and accessibility of suitable local data to support HTA varied widely but was mostly considered inadequate and limited.	Cross-sectional study	Nigeria
Workie, 2018 [30]	Ministry of Finance.	Health insurance, provider–funder separation.	To trace the factors that facilitated UHC success in the Seychelles, including high political commitment, strong voice and a downward accountability culture, strong public health functions, and an impressive investment in primary healthcare.	Two decades later, in 2000, the number of health centers more than doubled to 17; the number of doctors and dentists increased more than fivefold to 119; coverage of essential child, maternal, and reproductive health pushed to the upper 90%; the infant mortality rate dropped by over three quarters from 43.2 per 1000 live births in 1977 to 9.9 per 1000 live births in the year 2000; and average life expectancy for both sexes increased from 69.5 in 1980 to 72.7 years in 2000.	Cross-sectional study	Seychelles
Yip, 2001 [31]	Households.	Health insurance system that targets school children, School Health Insurance Programme (SHIP).	To assesses the extent to which the School Health Insurance Programme achieves its stated goals, that is, improving access and equity in access to healthcare for children.	Our findings show that the SHIP significantly improved access by increasing visit rates and reducing financial burden of use (out-of-pocket expenditures). Regarding the success of targeting the poor, conditional upon being covered, the SHIP reduced the differentials in visit rates between the highest- and lowest-income children. However, only the middle-income children benefited from reduced financial burden.	Cross-sectional study	Egypt
Koon, 2019 [32]	Different political contexts.	Evidence on the implementation of health systems strengthening (HSS) interventions is scarce. Donors need this information to prioritize investments and lobby for continued financial support.	To identify and reflect upon the factors that facilitate or impede health system strengthening interventions within and between contexts, and especially in donor-funded projects.	As donors’ transition to new forms of technical assistance, this article explores how attenuated contributions may continue to facilitate health system strengthening. This includes a strong commitment to participatory processes of engagement in the design and implementation of well-defined activities at multiple levels of the health system, by harnessing the power of an array of actors. We introduce a number of key considerations for donors, including the way in which HSS projects are structured, supported, and financed. The research presented here not only contributes to the global pool of knowledge on HSS, but also helps further implementation science, and thereby carries the potential to ultimately enhance health service delivery for the poor and vulnerable.	Cross sectional study	Ethiopia, Rwanda, and Zambia
Demissie, 2020 [33]	Household heads.	Community-based health insurance.	To analyze the effects of a community-based health insurance scheme on the utilization of healthcare services in Yirgalem town, southern Ethiopia.	The study reveals that community-based health insurance member households were about three times more likely to utilize outpatient care than their non-member counterparts.	Cross-sectional study	Ethiopia
Nelissen, 2020 [34]	Individuals with access to a private subsidized health insurance program.	Healthcare insurance program.	To explore the utilization of different types of healthcare providers and the factors associated with provider choice by insurance status in rural Nigeria.	The study shows that higher utilization of formal healthcare for insured compared to uninsured health episodes was driven by higher utilization of upgraded facilities. Upgraded facilities in the Kwara State Health Insurance Programme were visited during 20% of insured episodes compared with only 3% of uninsured episodes. Contrary to program implementers’ expectations, individuals with an uninsured episode did not access the upgraded facilities to benefit from the improved quality care.	Cross-sectional study	Nigeria
Woldemichael, 2019 [35]	Population-based analysis of district-level.	Trend of inequalities in accessibility of health center-based primary healthcare (PHC).	To assess availability and measure magnitude and trend of inequalities in accessibility of health center-based primary health care resources in Ethiopia from 2015 to 2017.	This analysis provided a clear picture of availability and inequalities in PHC resources across three regions in Ethiopia. Identifying contributing factors to low densities and high inequalities of SHWs may help improve PHC services nationwide, along with pathway toward UHC.	Cross-sectional study	Ethiopia
Were, 2020 [36]	Pregnant women living with HIV.	Effect of health insurance on obstetric healthcare.	To analyze the effect of health insurance on obstetric healthcare access including institutional delivery and skilled birth attendants for HIV+ pregnant women in Kenya.	This study confirms conceptual and practical considerations around health insurance and healthcare access for HIV+ persons. Further, it helps to inform relevant policy development for health insurance and HIV financing and delivery in Kenya and in similar countries in sub-Saharan Africa in the universal health coverage (UHC) era.	Cross-sectional study	Kenya
Sanogo, 2020 [37]	Antenatal care (ANC) visits during pregnancy, place of birth delivery, and postnatal health care.	Association of compulsory health insurance on maternal healthcare utilization.	To explore the wealth-related association of compulsory health insurance on maternal healthcare utilization in Gabon.	The findings proved that getting women to enroll in health insurance is a major strategy to improve the utilization of some important maternal health services, which include adequate ANC visits, delivery at health facilities, as well as attending postnatal care clinic. Generally, the findings of this study point to the fact that there is a significant increment in the use of standards recommended for adequate maternal healthcare which can be attributable to coverage of health insurance.	Cross-sectional study	Gabon
Mwase, 2018 [38]	Poor women who had completed a pregnancy in the 24 months prior to the survey date.	Inequities in access to and utilization of maternal care.	To assess the magnitude of the inequities and their determinants in coverage of maternal health services in Burkina Faso.	The study shows that existing inequities in maternal health services in Burkina Faso are likely going to jeopardize the achievement of Universal Health Coverage. It is important that policy makers continue to strengthen and monitor the implementation of strategies that promote proportionate universalism and forge multi-sectoral approach in dealing with social determinants of inequities in maternal health services coverage.	Cross-sectional study	Burkina Faso
Abiiro, 2014 [39]	Community residents.	Free access to an essential health package (EHP).	To explore how rural communities experience and define gaps in universal health coverage in Malawi, a country which endorses free access to an essential health package (EHP) as a means toward universal health coverage.	The study findings show that moving toward UHC in Malawi implies the introduction of appropriate interventions to fill the financial protection gaps in the private sector and the access-related gaps in the public sector and/or an effective public-private partnership that completely integrates both sectors.	Cross-sectional study	Malawi
Suchman, 2020 [40]	Exiting patients at health facilities.	Social health insurance.	To examine how social health insurance affects patient decision making regarding when and where to seek care in Kenya and Ghana.	The study shows that clients and providers would benefit from education on what is included in the SHI package. Providers should be monitored and held accountable for charging clients inappropriately; in Ghana, this should be accompanied by reforms to make government financing for SHI sustainable. Since clients value provider proximity and both Kenya and Ghana have a dearth of providers in rural areas, both countries should incentivize providers to work in these areas and prioritize accrediting rural facilities into SHI schemes to increase accessibility and reach.	Cross-sectional study	Kenya and Ghana
Knoblauch, 2019 [41]	Governments, implementers, drone providers, and funders of healthcare.	The use of bi-directional transport drones for health systems.	To perform a SWOT analysis (strengths, weaknesses, opportunities, and threats) use of bi-directional transport drones for health systems in sub-Saharan Africa.	Governments should also employ a system-strengthening approach to identify health system bottlenecks and explore new areas for supply chain optimization and cost-effectiveness using drones. After approximately 3 years of implementation of several proof-of-concept drone projects, the technology is still in its feasibility phase for many use cases. To this date, projects have yet to produce enough data to demonstrate a direct or indirect impact on health outcomes.	Case study	Madagascar, Malawi, and Senegal
Matthews, 2019 [42]	Health system governance and community health worker programs.	Strengthening the evidence ecosystem (using evidence to strengthen health system governance and support for community health worker programs; managing the growing epidemic of drug-resistant tuberculosis; social protection; the child support grant and its impact on health).	To apply an evidence ecosystem lens to the SA health system, and discuss its current functioning in support of the achievement of a high-quality health system that is able to achieve universal health coverage.	The evidence ecosystem model for health systems illustrates how evidence needs to be transferred between different key stages to strengthen health systems and inform care. It shows the importance of “closing the loop” between evidence producers, synthesizers, and disseminators and users.	Case study	South Africa
Miot, 2017 [43]	Public and private sector.	Optimization of pharmacoeconomics as a steering tool under the universal health coverage.	To explore factors that determine the introduction of pharmacoeconomics into health systems’ regulatory frameworks. Simultaneously, it seeks to provide guidance on processes for the design, implementation, and optimization of pharmacoeconomics as a steering tool within a health system under the UHC paradigm.	In the public sector, the process for selection of medicines onto the Essential Medicines List (EML) allows for the use of pharmacoeconomics to assess the cost-effectiveness of proposed additions to the list. This has developed over time, however, with earlier editions of the EML only considering clinical evidence and some costing. Even in the current environment, full pharmacoeconomic analysis is lacking in areas.	Case study	South Africa
Van Der Heever, 2012 [44]	Private health systems.	Health insurance.	To examine whether private health systems are susceptible to regulation and therefore able to support an extension and deepening of coverage when complementing a pre-existing publicly funded and delivered health system.	The private health system in South Africa has played an important supplementary role in achieving universal coverage throughout its history, but more especially in the post-Apartheid period. However, the quality of this role has been erratic, influenced predominantly by policy vacillation.The private system expanded rapidly during the 1980s mainly due to the pre-existence of a mature health insurance system and a weakening public hospital system that could accommodate and facilitate an increased demand for private hospital services.	Case study	South Africa
Mbogo, 2016 [45]	Males and females aged 23 to 59 years.	Financing population-based healthcare interventions.	To explore perspectives on employed individuals regarding financing population-based healthcare interventions toward UHC to make recommendations to the Ministry of Health on health financing options to cover population-based health services.	The study highlights that increasing enrolment in health coverage schemes requires targeted campaign for information dissemination through use of myriads mass media including: social networks, TV, radio and others. Moreover, re-designing health insurance schemes is critical in order to include population-based interventions and expand uptake of unemployed and informal sector employees; flexibility in monthly premiums payment plan; and use of technology to increase access to payment points.	Qualitative study	Botswana
Onarheim, 2018 [46]	Household members, health workers, and community members.	Intra-household resource allocation, focusing on how families prioritize newborn health and household needs in Ethiopia.	To explore intra-household resource allocation, focusing on how families prioritize new born health versus other household needs and their coping strategies for managing these priorities.	The study highlights that while improving neonatal health is prioritized at policy level in Ethiopia, poor households with sick neonates may prioritize differently. With limited money at hand and high direct health care costs, families balanced conflicting concerns to newborn health and family welfare. We argue that families should not be left in situations where they have to choose between survival of the newborn and economic ruin. Protection against out-of-pocket spending is key as Ethiopia moves toward universal health coverage. A necessary step is to provide prioritized newborn health care services free of charge.	Qualitative study	Ethiopia
Lu, 2012 [47]	Enrollees’ medical care utilization.	Health financing through Mutuelles community-based health insurance program.	To evaluate the impact of Mutuelles on achieving universal coverage of medical services and financial risk protection in its first eight years of implementation.	The findings show that Mutuelles improved medical care utilization and protected households from catastrophic health spending.	Quantitative	Rwanda
Mills, 2012 [48]	Households.	Healthcare financing.	To present a body of research whose overall aim was to critically evaluate existing inequities in health care financing and provision in Ghana, South Africa and Tanzania, and the extent to which health insurance mechanisms (broadly defined) could address financial protection and equity of access challenges.	Direct taxes were progressive in all three countries. Indirect taxes were regressive in South Africa but progressive in Ghana and Tanzania. Out-of-pocket payments were regressive and overall healthcare financing was progressive in all three countries. All forms of indirect tax (value-added tax (VAT), fuel levies, and excise duties) were regressive in South Africa. By contrast, VAT and excise and import duties were all progressive in Tanzania.	Qualitative study	Ghana, South Africa, and Tanzania
Foster, 2018 [49]	Nurse facility heads and district nurse supervisors.	Improving leadership capacity and management skills of facility heads.	To design and test a 12-month blended learning program for a certificate in leadership and management practice to build leadership and management competencies of rural facility heads, including increasing their ability to lead frontline teams and strengthening their skills and confidence in technology use.	Findings suggested that the facility heads had successfully strengthened their leadership and management competencies, increased their ability to lead frontline teams, and strengthened their skills and confidence in use of technology, including using a WhatsApp community of practice for support and consultation with other colleagues, with demonstrated improvements in the quality and accessibility of services.	Qualitative and quantitative study	Zambia

**Table 4:** Number of UHC studies conducted in Africa.

Country	Number of UHC Studies	References
Nigeria	6 studies	[17,18,26,29,34,50]
Ethiopia	5 studies	[21,32,33,35,46]
South Africa	5 studies	[26,42,43,44,48]
Tanzania	5 studies	[19,22,26,27,48]
Ghana	4 studies	[26,40,48,51]
Malawi	3 studies	[25,39,41]
Rwanda	3 studies	[26,32,47]
Kenya	2 studies	[36,40]
Senegal	2 studies	[24,41]
Zambia	2 studies	[32,49]
Algeria	1 study	[26]
Botswana	1 study	[45]
Burkina Faso	1 study	[38]
Egypt	1 study	[31]
Democratic Republic of Congo	1 study	[20]
Gabon	1 study	[37]
Madagascar	1 study	[41]
Seychelles	1 study	[30]
Zimbabwe	1 study	[28]
47 countries in the WHO African region	1 study	[23]

## Data Availability

The data generated from this paper will be made publicly available.
